# Sound insulation dataset of 30 wooden and 8 concrete floors tested in laboratory conditions

**DOI:** 10.1016/j.dib.2023.109393

**Published:** 2023-07-09

**Authors:** Valtteri Hongisto, Reijo Alakoivu, Juho Virtanen, Jarkko Hakala, Pekka Saarinen, Johann Laukka, Andreas Linderholt, Jörgen Olsson, Kirsi Jarnerö, Jukka Keränen

**Affiliations:** a Turku University of Applied Sciences, Acoustics Laboratory, Joukahaisenkatu 7, FI-20520 Turku, Finland; b Linnaeus University, Department of Mechanical Engineering, 351 95 Växjö, Sweden; c RISE Research Institutes of Sweden, Building Technology, 352 52 Växjö, Sweden

**Keywords:** Impact sound insulation, Airborne sound insulation, Wooden floors, Concrete floors, Cross-laminated timber, Open box timber

## Abstract

In a Finnish-Swedish consortium project, a large amount of sound insulation tests was conducted for several intermediate floors in laboratory conditions to serve various scientific research questions. The dataset contains 30 wooden and 8 concrete constructions which are commonly used between apartments in multistorey buildings. Impact sound insulation was determined according to ISO 10140-3 standard using both tapping machine and rubber ball as standard sound sources. Airborne sound insulation was determined according to the ISO 10140-2 standard. The data are special since they have a broad frequency range: 20−5000 Hz. Data are reported in 1/3-octave frequency bands and the single-number values of ISO 717-1 and ISO 717-2 are also reported. Detailed construction drawings are available for all reported constructions. The data are highly valuable for research, education, and development purposes since all data were obtained in the same laboratory (Turku University of Applied Sciences, Turku, Finland), and all the constructions were built by the same installation team.

**Specifications Table**

**Table:** 

Subject	Materials Science Engineering

Specific subject area	*Building acoustics; Airborne Sound insulation; Impact sound insulation; Floor constructions; Concrete floor; Timber floor;*
Type of data	Table Figure
How the data were acquired	The sound insulation data of the floors were obtained in controlled laboratory conditions where the structural flanking transmission was prevented. The data describes the inherent properties of floors. The floor area was 10.2 m^2^. Airborne sound insulation was measured according to the ISO 10140-2 standard. Impact sound insulation was measured according to the ISO 10140-3 standard using both a tapping machine and a rubber ball. Single-number values were determined according to ISO 717-1 and ISO 717-2. Acoustic measurements were conducted using a microphone. Data are based on one-third octave band frequency analysis.
Data format	Raw
Description of data collection	The data stem from measurements of 30 wooden and 8 concrete floor constructions. The area of each floor was 10.2 m^2^. The floors were built and measured in a standardized sound insulation laboratory. Floor constructions represent typical constructions used in apartment buildings. Floors were designed by researchers and personnel from industry. Floors were decided and mounted by the researchers.
Data source location	Turku University of Applied Sciences Acoustics Laboratory Joukahaisenkatu 7 FI-20520 Turku Finland
Data accessibility	Repository name: Mendeley Data Data identification number: Not available Direct URL to data: https://data.mendeley.com/datasets/y83p8mpryd/2

## Value of the Data

• The number of tested floors was exceptionally large, 38 floors.
• All floors were tested in full-scale size using a 10.2 m^2^ floor size.
• All floors were installed by the same team, which increases the reliability and comparability.
• The data can be used for the validation of sound insulation prediction models, education, and product development.
• The data can be benefited by material manufacturers, construction engineers, acoustic designers, building acoustics researchers, product and concept developers, and teachers in various levels of education.

## 1 Objective

The main purpose of the research project was to obtain reliable, measured sound insulation data [1] for wooden and concrete constructions that could be used for different scientific purposes. In overall, 38 full-scale floors were built to the sound insulation test laboratory and their impact and airborne sound insulation were determined by acoustic measurements using established standards [2], [3], [4], [5]. Laboratory testing of floors is expensive due to the material costs and large building efforts of full-size, load-bearing floors. Therefore, global sharing of the data is highly justified. In global level, most of the sound insulation laboratory tests are ordered by industrial companies for specific floors because the tests are expensive. Most of this private-owned data are never published by the companies. Opposite to that, the tests of our study were conducted for academic purposes. The sound insulation data now shared in Mendeley is highly valuable for professionals who do not have their own laboratory facilities and who need reliable experimental data.

## 2 Data Description

The dataset contains 30 wooden and 8 concrete floor constructions which are commonly used in multistorey apartment buildings. The impact sound insulation was determined according to the ISO 10140-3 [2] standard using both tapping machine and rubber ball as standard sound sources. The airborne sound insulation was determined according to the ISO 10140-2 [4] standard. The data are special since they span a broad frequency range: 20−5000 Hz. The data are reported in 1/3-octave bands and the single-number values of ISO 717-2 [3] and ISO 717-1 [5] are also reported. Detailed construction drawings are available for all reported constructions.

The Mendeley data [1] contains four files:

• Microsoft Excel file “**TUAS2023FloorSoundInsulationDataR1.xlsx**” contains the tabulated sound insulation values for 38 floors within the frequency band 20−5000 Hz.
• PDF file “**TUAS2023FloorConstructionDrawingsR1.pdf**” contains the detailed construction drawings of the 38 floors, their material listings, and the dynamic stiffnesses of the resilient layers used in the floating floors.
• ZIP file “**TUAS2023FloorProductSpecsR1.zip**” containing manufacturer's public data sheets concerning the materials. This file represents supplementary data, and it does not contain scientific data. Unfortunately, some of the sheets were not available in English.
• PDF file “**TUAS2023FloorDetails**” containing additional information about the construction of the floors. This file represents supplementary data, and it does not contain scientific data.

**Fig. 1:**
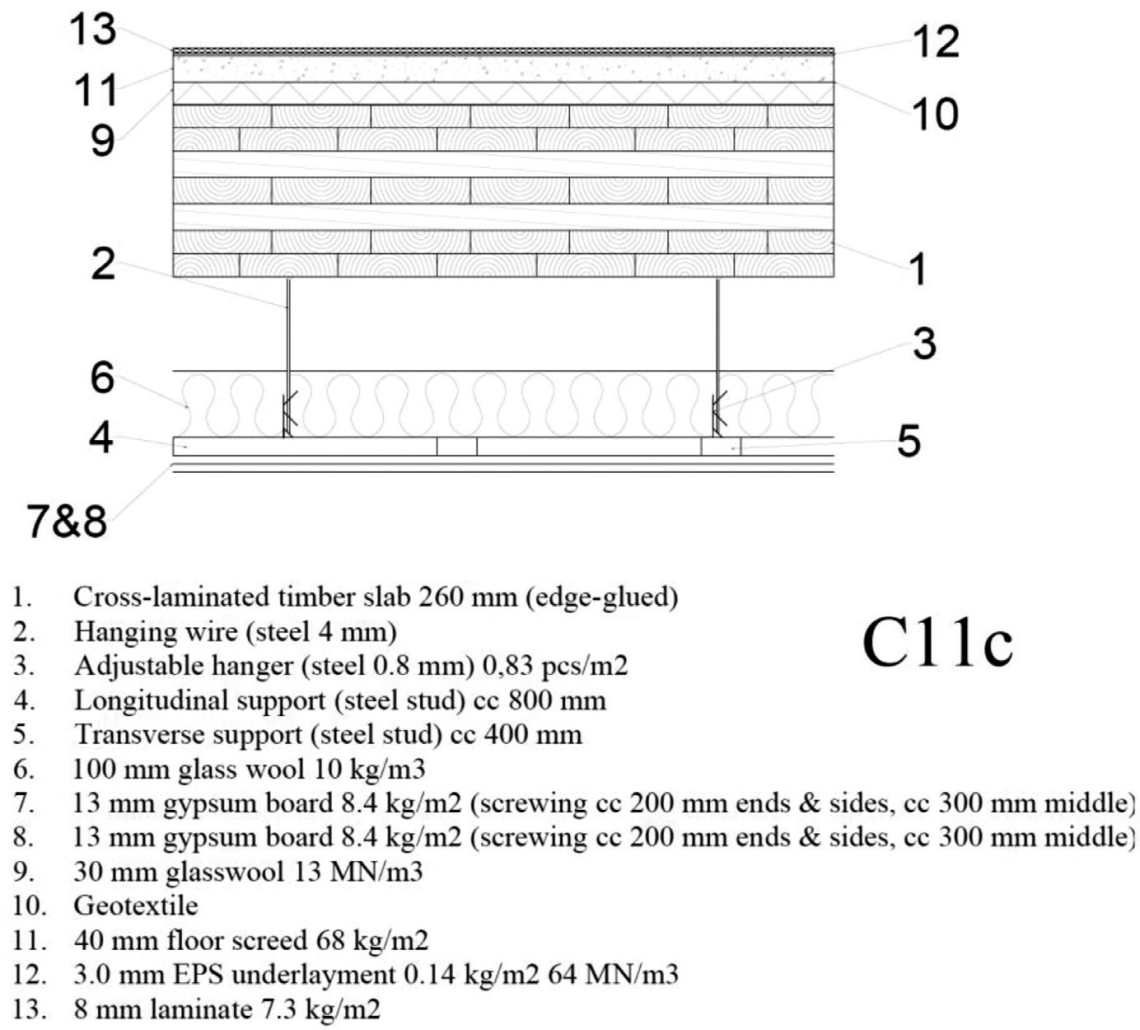
An example of the construction drawings (section drawings) available for each floor construction. This is the floor construction C11c.

**Fig. 2:**
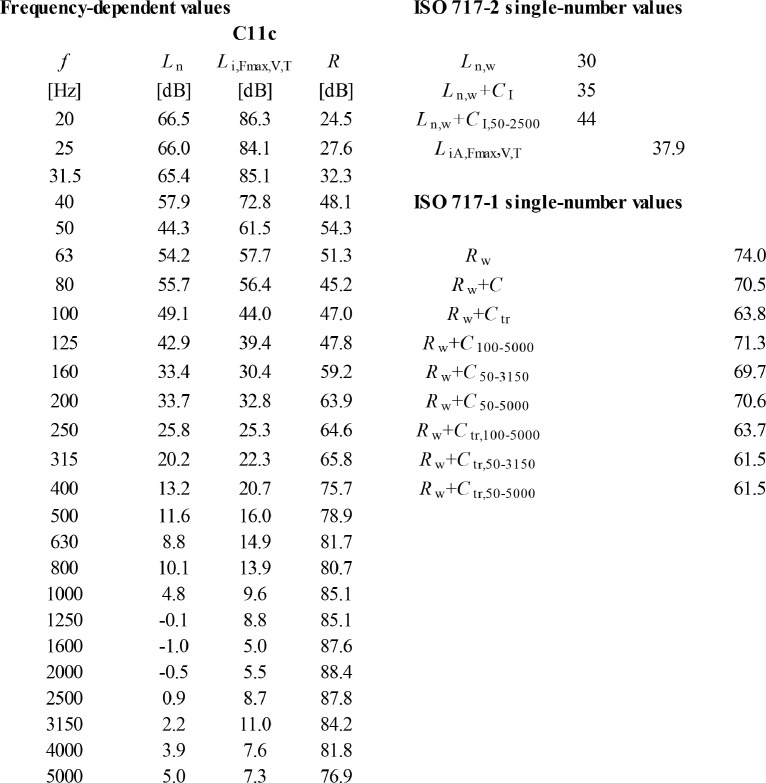
An example of the numerical sound insulation data available in Mendeley data [1] for each floor construction. This exemplary data concerns floor construction C11c.

The form of the numerical sound insulation data is depicted in Figs. 1−2 for one floor construction. All 38 floor constructions are reported in Ref. [1] using similar form.

## 3 Experimental Design, Materials and Methods

### 3.1 Floor Constructions

The sound insulation measurements were conducted for 38 floor constructions. They are described in detail in Mendeley Data file **TUASFloorConstructions2023.pdf** [1].

**Fig. 3:**
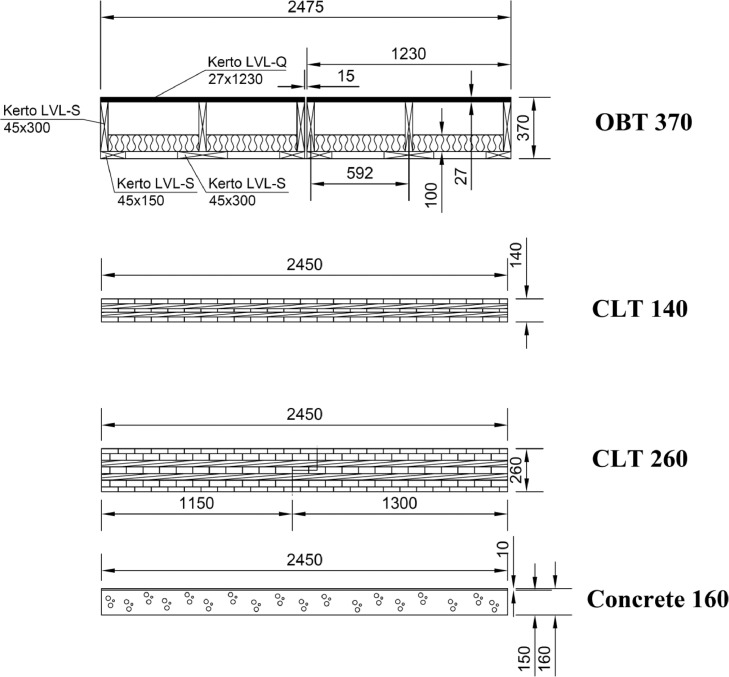
The four load-bearing slabs used in the measured floor constructions. OBT slab 370 mm (R). CLT slab 140 mm (X), CLT slab 260 mm (C). Steel-reinforced concrete slab 160 mm (H). The capital letters in brackets are used in the floor construction names of the data.

The measured floor constructions were based on three types of load-bearing slabs. They are clarified in Fig. 3. Bare load-bearing slabs cannot be used as such between residential dwellings since their sound insulation does not fulfill the sound insulation requirements of most countries. Therefore, the tested floor constructions had at least one kind of floor covering added to the load-bearing slab. In addition, suspended ceiling was included in several floor constructions. The load-bearing slab types and the number of different floor constructions tested for each slab type is depicted in Table 1.

**Table 1:** The three load-bearing slabs used in the floor constructions, the number of sound insulation tests available for each slab type, and the ranges of weighted normalized impact SPL, *L*_n,w_, and weighted sound reduction index, *R*_w_.

Type of load-bearing slab	Thickness [mm]	No. of floors	*L*_n,w_ [dB]	*R*_w_ [dB]
Open box timber slab	370	15	39−72	49−75
Cross-laminated timber slab (CLT)	140	4	50−70	38−55
Cross-laminated timber slab (CLT)	260	11	24−65	42−75
Steel-reinforced concrete slab	160	8	35−67	49−66

*Open box timber (OBT) slab* consisted of two 1230 × 4070 mm elements, which were mounted side-by-side with a 15 mm separation slit. The slit (4100 × 370 × 15 mm) was entirely sealed with mineral wool. The elements were tightened together (remaining the slit) with 300 mm screw distance using 200 × 5 mm cylinder-based wood screw. Screwing between the two elements was made alternately between opposing elements in 45 deg angle towards the opposing element. Kerto LVL is produced from 3 mm thick rotary-peeled strength graded softwood veneers (mainly spruce). The mean density is 510 kg/m^3^. The OBT slab thickness delivered from the factory was 370 mm thick. However, we had to nail 45 mm wood studs (cc400 mm) below the OBT slab. Therefore, the actual thickness of the OBT slab was 415 mm.

*CLT 260 slab* consisted of two 4070 × 1310/1160 mm elements, which were mounted side by side and pushed together (jaw joint). The elements were tightened vertically together using 240 × 7 mm broad-based wood screw with 300 mm screw distance. The slab consisted of 7 layers of spruce boards glued together (designation V7). Layers 3 and 5 were perpendicular to the other layers. The layer thickness was 30 mm in the surfaces and 40 mm elsewhere (30-40-40-40-40-40-30). The boards were edge-glued on all six edges, including the vertical board ends. Finger joints were used in the board ends. Two outer layers were parallel to each other. The density of CLT is approximately 470 kg/m^2^ at 12% relative humidity.

*CLT 140 slab* consisted of two 2450 mm wide and 2100/1950 mm long elements, which were mounted side by side and pushed together (jaw joint). The elements were tightened vertically together using 120 × 7 mm broad-based wood screw with 300 mm screw distance. The slab consisted of 5 layers of spruce boards glued together (designation H5). The layer thickness was 30 mm except 20 mm in the middle layer (30-30-20-30-30). The boards were edge-glued on all six edges, including the vertical board ends. Finger joints were used in the board ends. Each layer was perpendicular to the nearest layer. The density of CLT is approximately 470 kg/m^2^ at 12% relative humidity.

*Concrete 160 slab* corresponds to the heavy reference slab described in ISO 10140-1 [5]. It is permanently installed to the test opening C of the laboratory. It consists of 150 mm steel-reinforced concrete slab and 10 mm self-leveling floor screed. The slab is normally used to test the reduction of impact sound pressure level of floor coverings and floating floors.

The dry parts of the floor constructions were mounted and disassembled by the laboratory staff. Pumpable screed was mounted by external contractors. Construction drawings were made by two authors and checked by two other authors.

### 3.2 Building Acoustics Laboratory

**Fig. 4:**
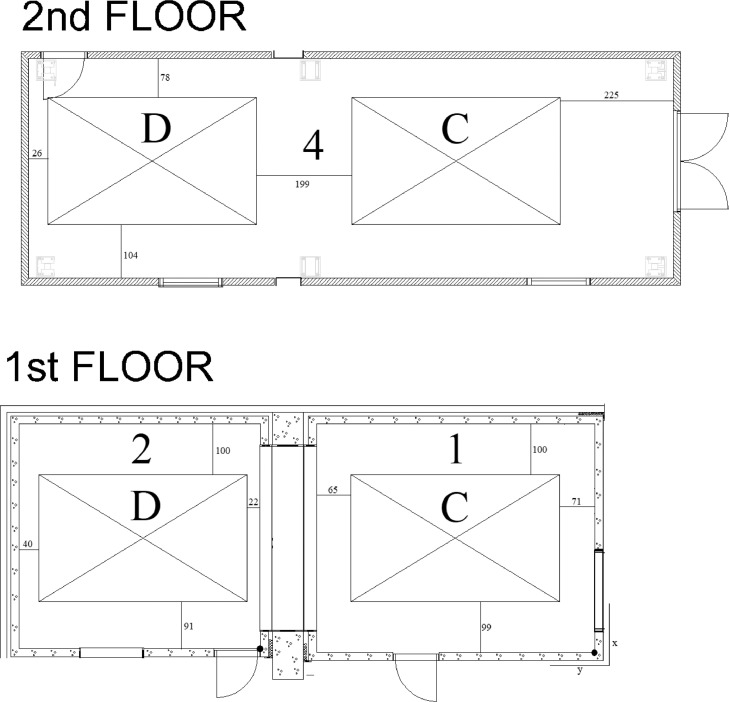
Layout of the sound insulation laboratory involving the floor test openings. Wooden floors were tested in test opening C. Concrete floors were tested in test opening D. The origo locates in the corner on floor height (black circle). Distances from test openings to room boundaries are given in centimeters. Numbers refer to Fig. 5.

**Fig. 5:**
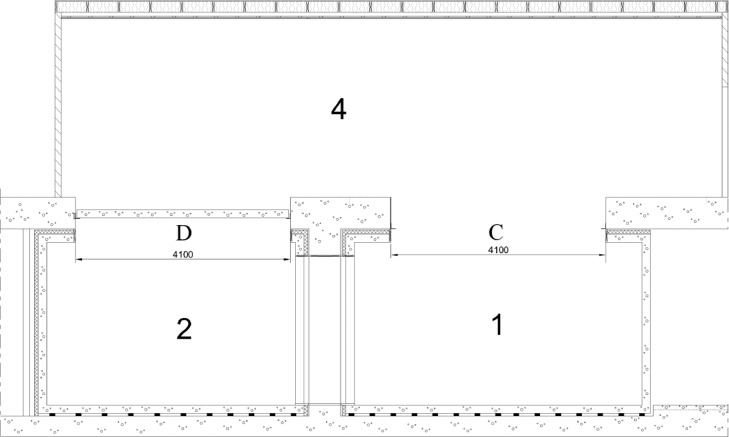
Section of the sound insulation laboratory. The test opening C does not contain a slab because three different slabs were investigated in that test opening. The test opening D contains the concrete 160 slab since it is permanent. The floor, wall, and ceiling ring of rooms 1−2 was 160 mm reinforced concrete. Rooms 1−2 were mechanically detached from building frame using polyurethane isolators under the floor.

**Fig. 6:**
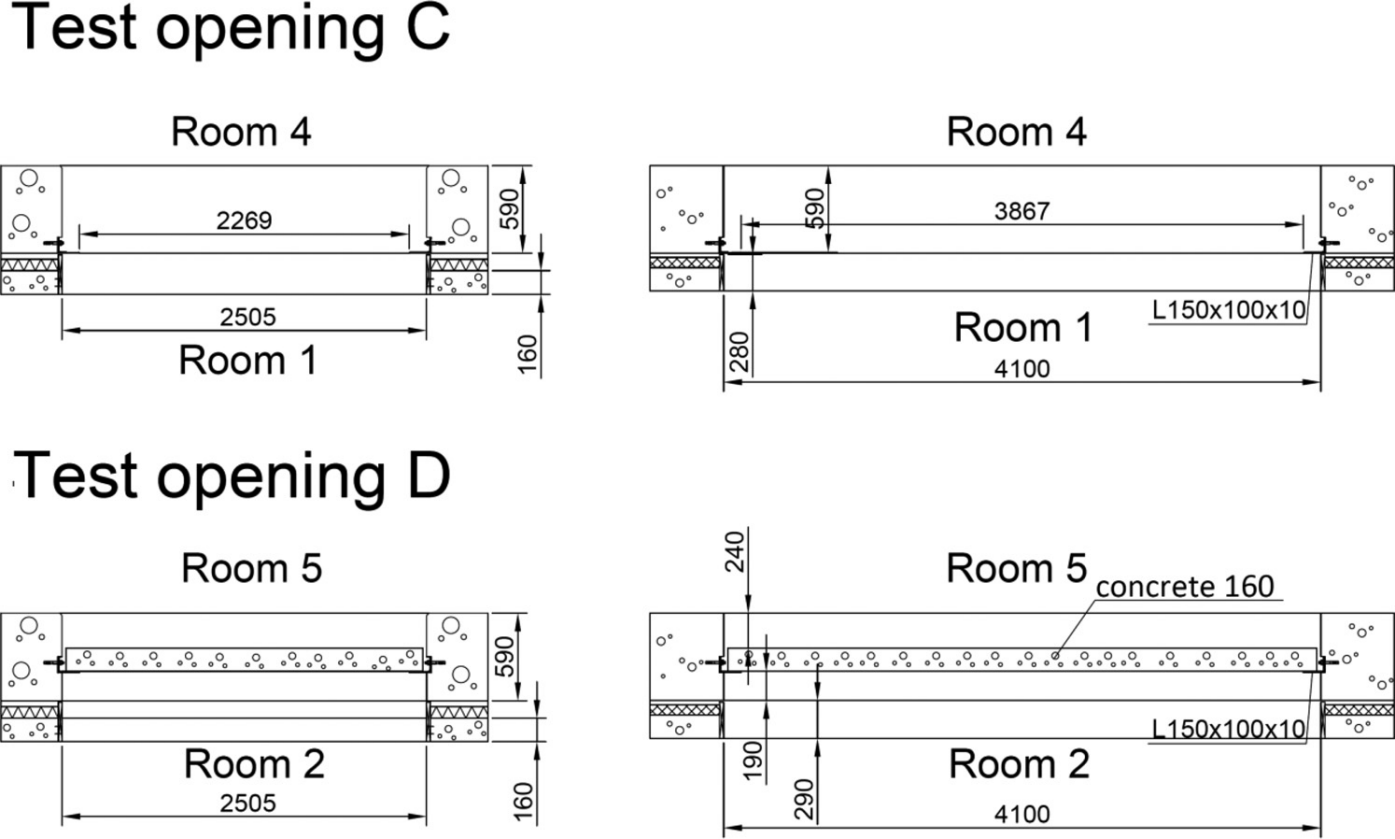
Dimensions of the test openings C and D. The 160 mm steel-reinforced concrete slab is permanently installed in the test opening D, and it is therefore shown. Instead, test opening C is empty, because various slabs can be installed into it. The slabs were installed on top of an L-shaped steel beam of size 150 × 100 × 10 mm.

Sound insulation measurements were conducted in the building acoustics laboratory described in Figs. 4−5. The dimensions of the test openings and test rooms are shown in Fig. 6 and Table 2. The laboratory fulfills the mandatory requirements of ISO 10140-1 standard for sound insulation test laboratories [6] within 50−5000 Hz. The laboratory was also an accredited test laboratory (according to ISO/IEC 17025:2017) to the measurements described in Sec. 2.3 and Sec. 2.4.1 within 50−5000 Hz. Accreditation was made by FINAS (Finnish Accreditation Service).

**Table 2:** Dimensions of the reverberant chambers of Fig. 5.

	Length	Width	Height	Volume	Floor area
Room	[m]	[m]	[m]	[m^3^]	[m^2^]
1	5.49	4.50	3.07	75.8	24.7
2	4.75	4.42	3.06	64.4	21.0
4	12.55	4.32	3.40	184.3	54.2

### 3.3 Airborne Sound Insulation

The airborne sound insulation was determined according to ISO 10140-2 [1]. The measurements were conducted between two adjacent rooms, A and B, which are separated by the floor construction being tested. In this case, room A was on top of room B since the investigation concerns horizontally mounted floor constructions. The rooms A and B were mechanically separated from each other so that structural flanking sound transmission was suppressed. Suppression was implemented by vibration isolators under room B (i.e., rooms 1 and 2).

The test noise is produced in room A and the sound pressure level, SPL, is measured in both the rooms, A and B. The physical quantity of airborne sound insulation is the **sound reduction index, *R*** [dB], determined by

(1) $$R=L_{A}-L_{B,c}+10\mathrm{lg}\frac{S}{A_{B}}$$

where *L*_A_ [dB] is the spatially averaged SPL of the test signal in the source room A, *L*_B,c_ [dB] is the background noise corrected SPL of the test signal transmitted to the receiving room B, *S* [m^2^] is the area of the floor (10.2 m^2^), and *A_B_* [m^2^] is the absorption area of room B. The absorption area is determined as

(2) $$A_{B}=0.16\frac{V_{B}}{T_{B}}$$

where *T*_B_ [s] and *V*_B_ [m^3^] are the reverberation time and volume of room B, respectively.

The background noise corrected SPL, *L*_B,c_ [dB], is determined from

(3) $$L_{B,c}\left\{ \begin{array}{cc} L_{B,tot}-1.3\;\text{dB}, & L_{B,tot}-L_{B,bg}<6\;\;dB\\ 10\cdot \log_{10}\left(10^{L_{B,tot}/10}-10^{L_{B.bg}/10}\right) & \;\;6\;\;dB\leq \;L_{B,tot}-L_{B,bg}\leq 15\;\;dB\\ L_{B,tot} & \;\;L_{B,tot}-L_{B,bg}>15\;\;dB \end{array}\right.$$

where *L*_B,bg_ [dB] is the SPL of the background noise in room B and *L*_B,tot_ [dB] is the total SPL produced by the background noise and the test signal transmitted room A measured in room B. Because background noise depended on the position, the correction was made separately for each measurement position.

Two setups of rooms were used in this investigation (see room numbers of Fig. 4):

• Concrete floors: the source room A was room 4 and the receiving room B was room 2;
• Wooden floors: the source room A was room 4 and the receiving room B was room 1;

Identical measurement apparatus was used in both setups.

The test signal was wide-band pink noise. It was produced to the source room using four loudspeakers (custom made: two middle frequency sources, one bass source, and one descant source). Independent pink noise generators were used for each loudspeaker (Behringer Ultra-Curve DEQ 2496, Behringer Ultra-Curve DEQ 2496, Behringer Ultra-Curve DEQ 2496, Behringer Ultra-Curve DEQ 2496). Three stereo amplifiers were used (Samson SERVO 260, QSC RMX 850, QSC RMX 850a).

The spatial average of the SPL was measured in both rooms using rotating microphone boom (Brüel&Kjær 3923). The rotating radius was 100 cm, and the measurement time was one rotation, lasting 64 s. The inclination was set so that the lowest and the highest positions of the microphone from the floor were 90 cm and 180 cm, respectively. The SPL measurements were conducted with condenser microphone (Brüel&Kjær 4165, preamplifier Brüel&Kjær 2669). Similar apparatuses were used in rooms A and B. The SPL measurements were conducted simultaneously in rooms A and B using a 2-channel real-time analyzer (Norsonic 121).

The reverberation time of room B was determined according to standard ISO 3382-2 [7] using the interrupted noise method. The test signal (pink noise) was produced in room B using real-time analyzer (Norsonic 121) and amplifier (QSC 900 W USA). Two fixed positions of omnidirectional loudspeakers and three fixed microphone positions were used. The microphones were the same as above, but the rotating boom movement was turned off, and the fixed microphone positions were adjusted manually. Two measurements were conducted for each loudspeaker-microphone position. The reverberation time was determined from 12 measurements using the decay time of 20 dB SPL reduction. A real time analyzer was used to determine the reverberation time (Norsonic 121).

The microphone and the analyzer were checked at 1 kHz before every measurement using constant sound source (Brüel&Kjær 4231). Microphone, analyzer, and constant sound source were traceably calibrated within the last 12 months in an accredited calibration laboratory.

The single-number values of airborne sound insulation (weighted sound reduction index, *R*_w_) and spectrum adaptation terms using spectrum *C* (4 alternatives) and *C*_tr_ (4 alternatives) were determined according to standard ISO 717-1 [2].

The standard [1] supports measurements only within 50−5000 Hz. However, we reported the values within 20−5000 Hz. The uncertainty within 20−40 Hz has not been determined and it is probably larger than within 50−80 Hz.

### 3.4 Impact Sound Insulation

**Fig. 7:**
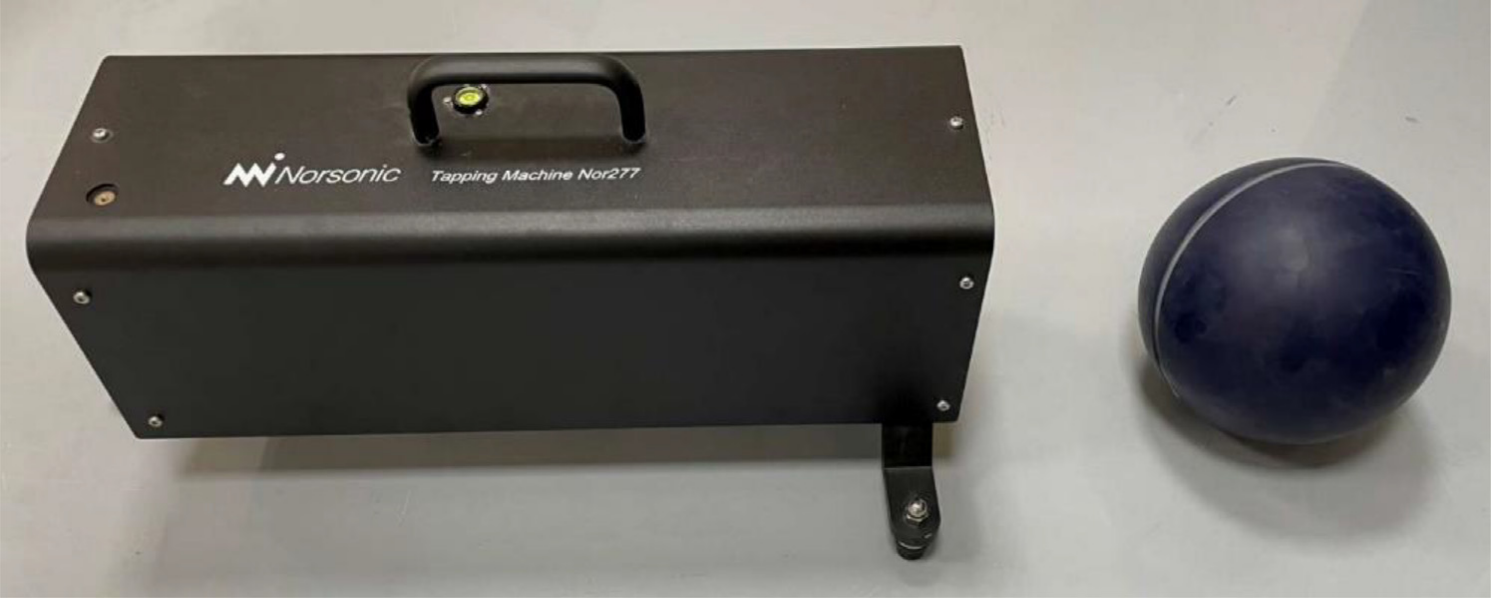
Investigated impact sound sources. Left) Tapping machine. Right) Rubber ball (heavy/soft impact source).

**Fig. 8:**
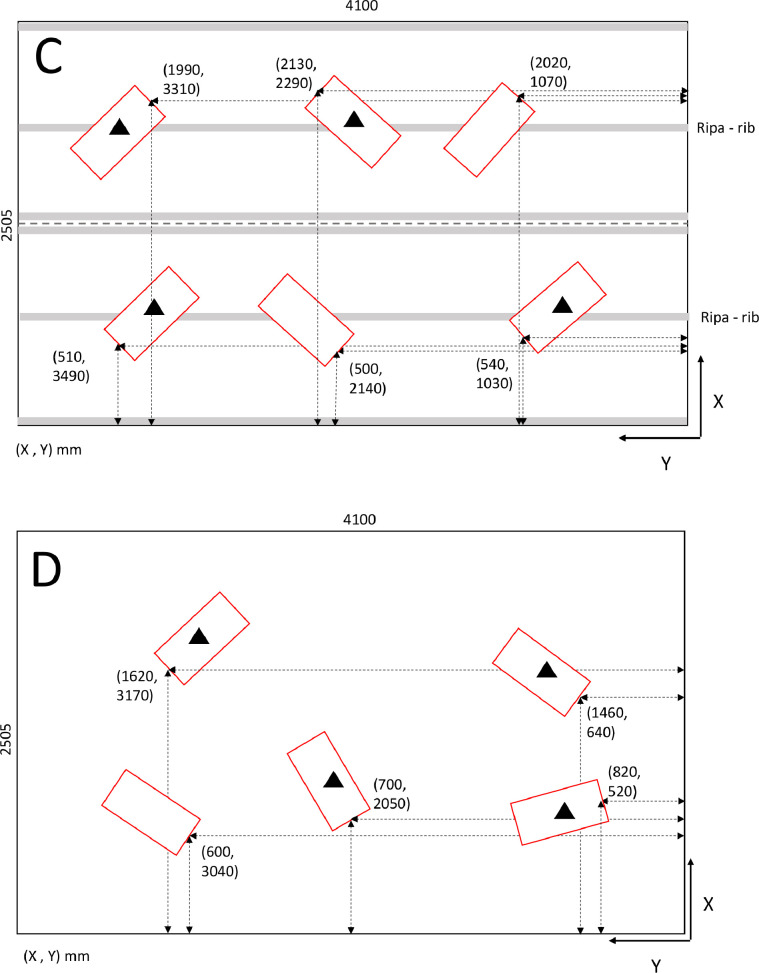
Positions of the tapping machine and impact ball drops in the test openings C and D and their distances with respect of origo of the test opening. The positions of the test openings conform with Fig. 4. Rib means the position of rib in OBT slab.

Impact sound insulation was determined according to ISO 10140-3 [3] in the same setup of the floor construction as in Sec. 2.3. The impact was made upstairs towards the floor in room A. All acoustic measurements were conducted in room B, downstairs, i.e., under the floor. The measurements were conducted using both impact sound sources according to the ISO 10140-4 standard, i.e., a tapping machine and a rubber ball [6], see Fig. 7. The positions of the impact sources in the slabs are shown in Fig. 8. According to the standard, the former simulates impact sources like human footsteps when a person is wearing shoes. The latter simulates impact sources with strong low frequency components, such as human footsteps (bare feet) or children jumping.

#### 3.4.1 Tapping Machine As The Impact Sound Source

**The normalized impact SPL, *L*_n_ [dB],** was determined as

(4) $$L_{n}=L_{B,c}+10\cdot \log_{10}\left(\frac{A_{B}}{A_{0}}\right)$$

where *L*_B,c_ [dB] is the background noise corrected SPL of the tapping machine in room B (see Eq. 3), *A*_B_ [m^2^] is the absorption area of room B (as in Eq. 2), and *A*_0_=10 m^2^.

The tapping machine (Norsonic 277) was placed on the floor in room A. Five positions of the tapping machine were used. The measurement apparatus was the same as in Section 3. Reverberation times of Sec. 2.3 were used to determine *A*_B_.

Tapping machine was traceably calibrated in an accredited calibration laboratory.

Single-number values of impact sound insulation (weighted normalized impact sound pressure level, *L*_n,w_), spectrum adaptation term *C*_I_ and spectrum adaptation term *C*_I,50-2500_ were determined according to the ISO 717-2 [2] standard.

The standard [3] supports measurements only within 50−5000 Hz. However, we reported the values within 20−5000 Hz. The uncertainty within 20−40 Hz has not been determined and it is probably larger than within 50−80 Hz.

#### 3.4.2 Rubber Ball As The Impact Sound Source

A rubber ball (Rion YI-01) was dropped from 1.000 m height from the floor level. The drop was made in the source room A in four positions (Fig. 8). For each position, the Fast-time weighted maximum level, *L*_F,max_, was measured in the receiving room B in four fixed microphone positions (Table 3) so that the total number of measurements was 16. The reverberation time determined in Sec. 2.3 was utilized. The average background noise corrected value is denoted as *L*_B,i,F,max,c_. The background noise correction was made as in Eq. 3.

**Table 3:** The coordinates of the microphone positions 1−4 in rooms 1 and 2 in centimeters. The xy-coordinates are shown in Fig. 4. Coordinate z is the height.

	Room 1				Room 2		
Position	x	y	z		x	y	z
1	115	145	110		320	375	202
2	325	200	130		102	303	170
3	255	425	170		340	115	215
4	160	295	150		246	268	101

The **standardized maximum impact SPL, *L*_i,Fmax,_*_V,T_* [dB],** was determined as

(5) $$L_{i,F\max,V,T}=L_{B,i,F\max}+10\cdot \log_{10}\left(\frac{V_{B}}{V_{0}}\right)-10\cdot \log_{10}\left[\left(\frac{1-\frac{1}{C_{0}}}{1-\frac{1}{C}}\right)\cdot \left(\frac{\frac{1}{C^{\left(1-C\right)}}-\frac{1}{C^{-\left(1-\frac{1}{C}\right)}}}{\frac{1}{C_{0}^{\left(1-C_{0}\right)}}-\frac{1}{C_{0}^{-\left(1-\frac{1}{C_{0}}\right)}}}\right)\right]$$

where *V*_B_ [m^3^] is the volume of the receiving room B, *V*_0_=50 m^3^, *C*_0_=0.2894, and

(6) $$C=\frac{T}{1.7275}$$

where *T*_B_ [s] is the reverberation time in the receiving room.

Measurements were conducted using a real time analyzer (Nor150) equipped with condenser microphone (Nor1225) and a preamplifier (Nor1209).

The real time analyzer was tested before every measurement using a constant sound source (Brüel&Kjær 4231). The real-time analyzer and the constant sound source were traceably calibrated in an accredited calibration laboratory within the last 12 months.

The single-number value (standardized weighted maximum impact sound pressure level, *L*_iA,Fmax,V,T_) was determined according to ISO 717-2 [2].

The standard [3] supports measurements only within 50−630 Hz. However, we reported the values within 20−5000 Hz. The uncertainty within 20−40 Hz has not been determined and it is probably larger than within 50−80 Hz.

### 3.5 Dynamic Stiffness

A large proportion of the measured floors involve a floating slab on top of the load-bearing slab. These slabs are separated by a resilient material layer. The dynamic stiffness per unit area for the resilient layer, *s*’ [Pa/m, N/m^3^], was determined according to the ISO 9052-1 [8] standard.

For each resilient material, three samples with a cross-sectional area of 200 × 200 mm were measured. The mean of the three samples is reported.

**Fig. 9:**
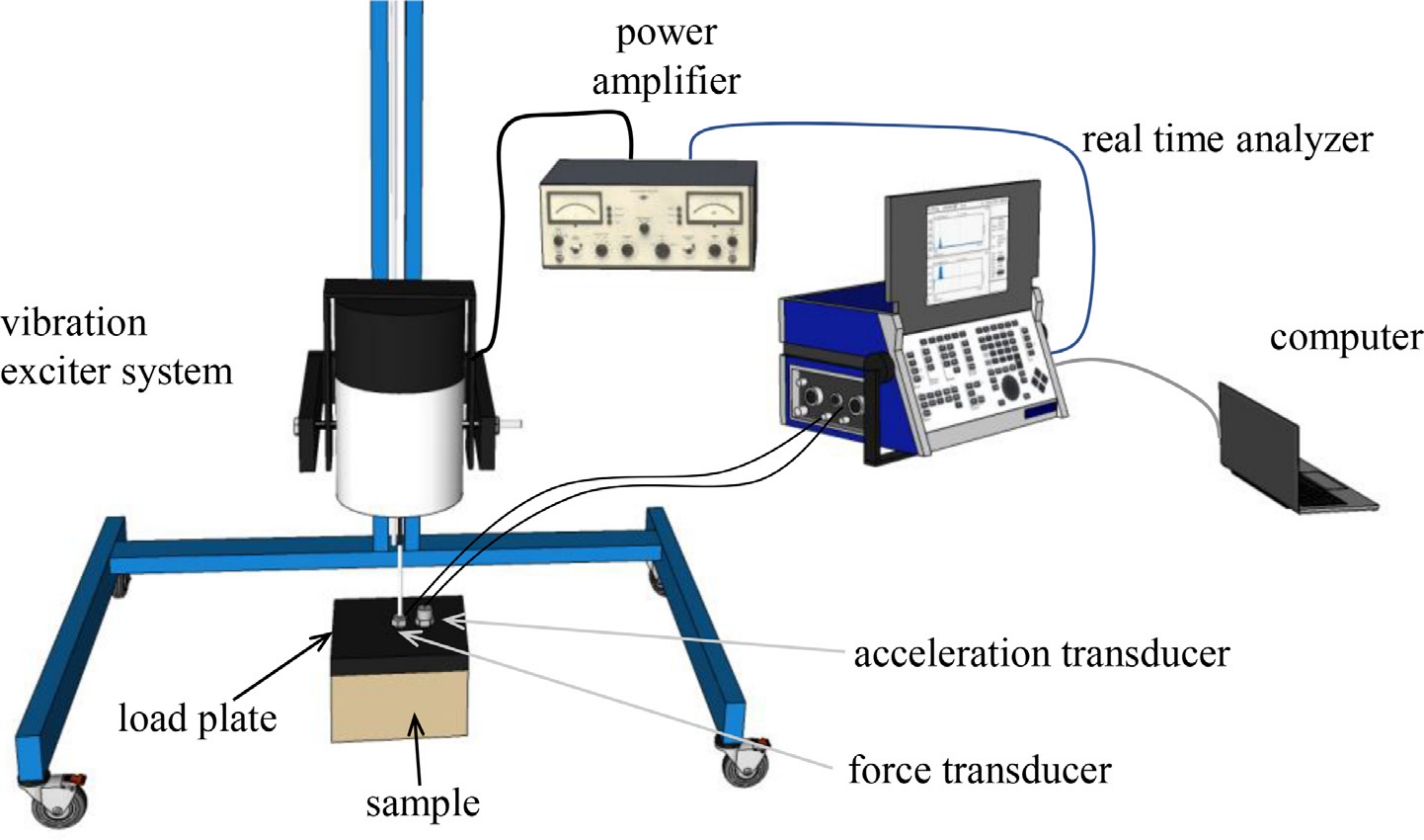
Determination of dynamic stiffness.

The measurement arrangement is shown in Fig. 9. The resilient sample was placed against smooth and heavy concrete floor (> 600 kg/m^2^, no resilient toppings). A rigid load plate (steel 25 × 200 × 200 mm, *m*_L_=7.8 kg) was placed on top of the sample so that the load plate, the resilient sample, and the floor formed a mass-spring system. The load plate was excited to vibrate using a shaker (Brüel&Kjær 4813) via a vertical rod between the shaker and the load plate. The force against the load plate is denoted by *F* [N] and it was measured with a force transducer (Brüel&Kjær 8200). The wide-band vibration signal was white noise. It was produced with the real-time analyzer (Norsonic 840A). The signal was then amplified (Brüel&Kjær 2707). The acceleration of the load plate, denoted by *a* [m/s^2^], was measured using an accelerometer (Brüel&Kjær 4370). The Fast Fourier Transform was used to determine the dependence of both *a* and *F* on frequency *f* [Hz]. The frequency resolution was 0.244−0.977 depending on the resonance frequency. Both *a*(*f*) and *F*(*f*) were determined using real-time analyzer (Norsonic 840A). Their ratio, i.e., the accelerance *A*(*f*) [1/kg], was determined from

(7) $$A\left(f\right)=\frac{a\left(f\right)}{F\left(f\right)}$$

**Fig. 10:**
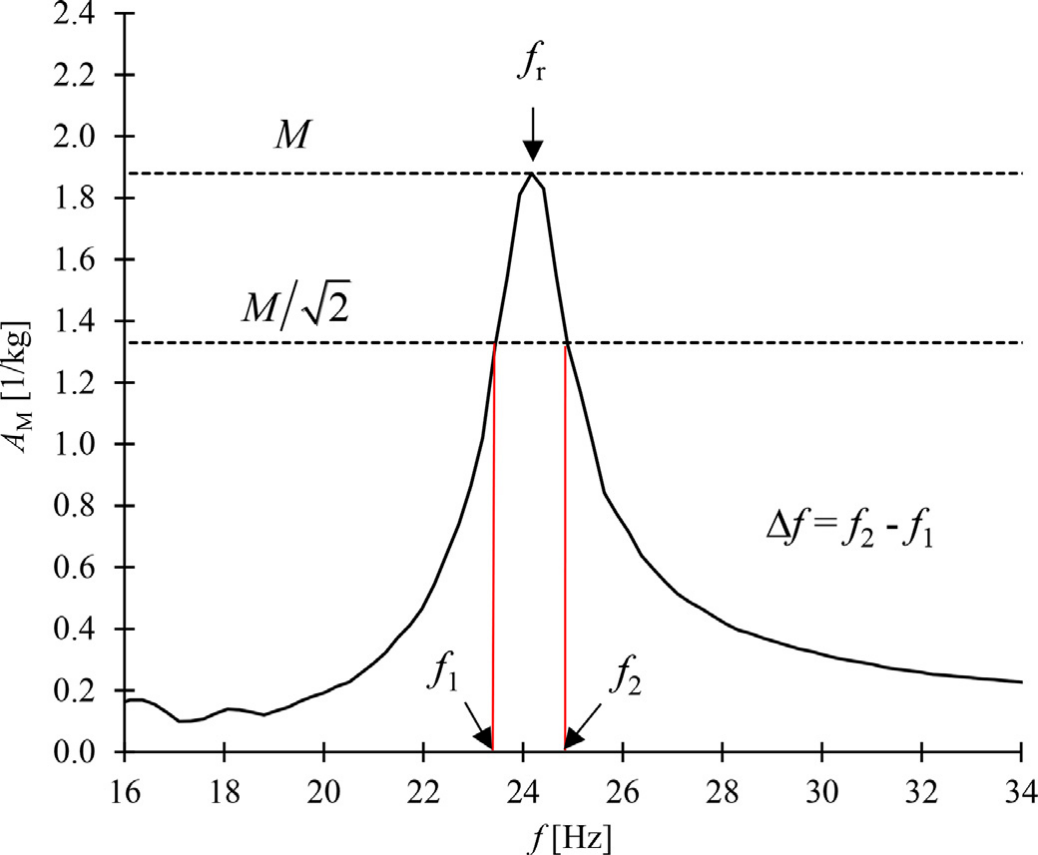
The determination of resonance frequency, *f*_r_, and half-power bandwidth, Δ*f*, from the accelerance magnitude, *A*_M_, versus frequency, *f*, curve. *M* means the accelerance magnitude at *f*_r_.

At the resonance frequency, *f*_r_ [Hz], the load plate vibrates strongly and the accelerance magnitude (*A* is a complex variable) reaches a local maximum value (resonance peak), which can be visually determined from the graphical data (Fig. 10). Apparent dynamic stiffness per unit area, *s*’_t_ [N/m^3^], was determined from

(8) $$s{{}^{\prime }}_{t}=4\pi^{2}m_{L}f_{r}^{2}/S$$

where *S* [m^2^] is the area of the sample (0.04 m^2^).

The dimensionless loss factor of the resilient material, *η*, describes the internal power losses of the material. It was determined according to the method proposed by Schiavi [9] using equation

(9) $$\eta =\frac{\Delta f}{f_{r}}$$

where Δ*f* [Hz] is the half power bandwidth of the resonance peak. Fig. 9 depicts the determination of both *f*_r_ and Δ*f*.

If the airflow resistivity of the resilient material, *r*, is large (>100 kPa⋅s/m^2^), the outcoming *s*’ is the same as *s*’_t_. Such materials are, e.g., EPS-based materials, where the air cannot freely flow through the material. On the other hand, if the airflow resistivity of the resilient material is low (e.g., wools), the stiffness of air contributes to the stiffness of the material, when the material is placed under the floating slab and air cannot escape from the material unlike in the test setup of Fig. 10. Then, the value of *s*’_t_ must be corrected by the dynamic stiffness of air per unit area, *s*’_a_ [N/m^3^], for a layer of thickness *d*. Therefore, the airflow resistivity of resilient materials was determined according to the ISO 9053-1 standard [[10], [11]].

Finally, the **dynamic stiffness per unit area, *s*’** [N/m^3^], was determined from

(10) $$s^{\prime }=s{{}^{\prime }}_{t}+s{{}^{\prime }}_{a}=s{{}^{\prime }}_{t}+\frac{p_{0}}{d\left(1-\rho /\rho^{\prime }\right)}$$

where *p*_0_ [Pa] is the atmospheric pressure of air (usually 101300 Pa), *d* [m] is the thickness of resilient material (and air layer), *ρ* [kg/m^3^] is the density of resilient material (including the air), and *ρ'* [kg/m^3^] is the density of the raw material of the resilient material (air pores are excluded).

ISO 9052-1 recommends that the correction for air stiffness in Eq. 10 is not made if airflow resistivity is beyond 10−100 kPa⋅s/m^2^.

## Ethics Statements

Neither human nor animal data is involved.

## CRediT authorship contribution statement

**Valtteri Hongisto:** Conceptualization, Project administration, Supervision, Funding acquisition, Investigation, Methodology, Resources, Validation, Visualization, Writing – original draft, Writing – review & editing. **Reijo Alakoivu:** Investigation, Data curation, Formal analysis, Methodology, Validation, Writing – original draft. **Juho Virtanen:** Investigation, Visualization. **Jarkko Hakala:** Investigation, Visualization. **Pekka Saarinen:** Investigation, Visualization, Writing – original draft. **Johann Laukka:** Investigation. **Andreas Linderholt:** Writing – original draft. **Jörgen Olsson:** Writing – original draft. **Kirsi Jarnerö:** Funding acquisition. **Jukka Keränen:** Methodology, Validation, Investigation.

## Declaration of Competing Interests

X The authors declare that they have no known competing financial interests or personal relationships that could have appeared to influence the work reported in this paper.

## Data Availability

TUAS 2023 Floor Sound Insulation Rev1 (Original data) (Mendeley Data).
